# The changing trends in tobacco smoking for young Arab women; narghile, an old habit with a liberal attitude

**DOI:** 10.1186/1477-7517-8-24

**Published:** 2011-08-30

**Authors:** Najla S Dar-Odeh, Osama A Abu-Hammad

**Affiliations:** 1 Department of Oral and Maxillofacial Surgery, Oral Medicine and Periodontics, Faculty of Dentistry, University of Jordan, Queen Rania Street, Amman, 11942, Jordan; 2 Department of Prosthodontics, Faculty of Dentistry, University of Jordan, Queen Rania Street, Amman, 11942, Jordan

**Keywords:** narghile, young Arab females, tobacco

## Abstract

Narghile smoking by young females is becoming more acceptable than cigarettes in the conservative societies of Arab countries. Lack of social constraints on narghile smoking has resulted in an increased prevalence of narghile smoking among young Arab females and an earlier age of onset of this habit when compared to cigarette smoking.

Documented health hazards of narghile smoking including pulmonary, cardiovascular and neoplastic ailments are consequently expected to affect this vulnerable sector of the population together with their offspring. In this commentary, we shed some light on the changing trend of tobacco use among young Arabic women as shown by an increasing number of studies investigating habits of tobacco use in young people.

## Prevalence of narghile smoking among young Arab females

Although cigarette smoking is the most common type of tobacco use worldwide, narghile smoking (NS) is spreading globally to affect Arabic and western societies. Recent studies have demonstrated a high prevalence of NS among females in many Arabic countries [1-5]. NS is one of the social habits that shape the general forms of Arabic folklore. It is considered socially acceptable by a substantial proportion of the society including young females. This lenient role of the society in accommodating or even encouraging females to smoke narghile, seems to override the efficient role of religion. Islam is the religion of most Arabs and it essentially demands paying attention to own health as well as public health [6]. Religious authorities and clerics emphasize this attitude towards NS to the public over the media and during Friday prayers. Among Muslim populations, smoking is considered inappropriate for girls, is unladylike, and may ruin a girl's reputation and prospects for marriage [7]. Hence, cigarette smoking has always been a limited or a concealed habit within the Arabic female population particularly unmarried ones. However, the trend of NS has changed the picture and Arabic females -whether married or not- can now smoke narghile in the open without feeling embarrassed or ashamed about it. This permissive role of the society also delivers a wrong message about narghile safety to the public. Many health professionals perceive smoking narghiles as being less harmful than cigarette smoking or even not harmful based on the presumption that the inhaled smoke is filtered through water [8].

Several studies conducted in most Arabic countries showed that NS is growing in popularity among young females, and trends have shifted between tobacco types, with NS becoming the preferred form of tobacco use for women [9].

Statistics for the prevalence of NS among young Arab females are disturbing (table-1) particularly in countries like Egypt [5], Lebanon [10,11], Jordan [2,3] and Syria [1]. Prevalence was also relatively high among Palestinian girls [4] and those in the gulf region [12]. Data from more conservative societies like those of Saudi Arabia were scarce as the studies conducted there investigated only males [12,13]. A recently published study, however, showed that about half of the smoker female students in a Saudi university smoke narghile, while the other half smoke cigarettes [14].

**Table 1:** Prevalence of narghile smoking among young females of the Arabic states

Researchers	Country	Age Category	Prevalence
Labib et al (2007)[5]	Egypt	University students	37.8%

Maziak et al (2004)[1]	Syria	University students	4.9%

Azab et al (2010)[2]	Jordan	University students	19%

Al-Mulla et al (2008)[12]	Gulf states	13-15 years	6.4%-12%

Mandil et al (2010)[14]	Saudi Arabia	University students	2%

Tamim et al (2003)[10]	Lebanon	University students	23.4%

Harrabi et al (2010)[15]	Tunisia	13-17 years	0.2%

With the exception of Tunisia [15] in which more girls in the age group of 13-17 years favor cigarettes (1.1%) over narghile (0.2%), the female preference for narghile is obvious, confirming the influential role society plays in shaping smoking behavior of young females.

## Risks of NS by young females

Studies investigating the effects of NS on health are increasingly reporting its adverse effects. The habit is associated with increased risk of chronic obstructive airway disease as well as adverse cardiovascular effects like increased blood pressure [16-19] There have also been reports of its association with some types of cancer such as bronchogenic carcinoma, oesophageal carcinoma, bladder cancer and pancreatic cancer [20-23] The reader can refer to a number of reviews written in this regards [24-27].

Moreover, one can not consider NS as a "safer" alternative to cigarettes when discussing the important issues of young age and female gender.

It is obvious from the studies investigating prevalence of NS in the Arabic countries that females opt for the narghile and they do so unacceptably at the young age of early adolescence. Exposure to any form of tobacco at young age is expected to increase the risk of tobacco-associated disease. A strong correlation seems to exist between incidence of oral cancer and the age of onset of tobacco use [28]. Consequently, a higher incidence of oral cancer in men and women of younger age group is seen in developing countries than in the United States for example [28].

Another important aspect pertaining to the young age of female narghile smokers is pregnancy. It was shown that smoking one or more narghiles a day during pregnancy is associated with at least a 100 g reduction in the adjusted mean birth weight of babies [8]. In addition, babies born to women smoking narghile during pregnancy have a higher proportion of other problems, such as pulmonary diseases [8]. Even for women who are ready to give up smoking when pregnant, some may not be aware of their pregnancy, and hence they continue smoking. Unfortunately, the risk of having babies of low birth weight almost triples among those who smoke narghiles in the first trimester [8].

## Café narghile smoking

**Figure 1:**
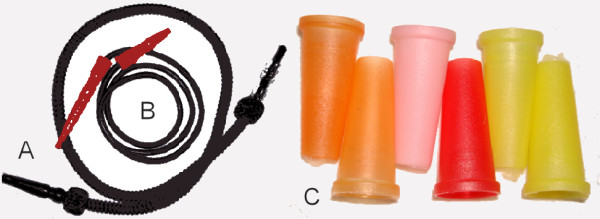
**Disposable parts of the narghile used in cafes: A, regular hose is understandably associated with cross infection**. B, disposable (hygienic) hose have less diameter making smoking less preferable. C, disposable tips (mabsams) made of plastic posing health hazards.

Young narghile smokers prefer usually to socialize with their friends in cafés during narghile smoking [3,5]. During these social gatherings many of them share one narghile set through using its hose tip, so that the narghile hose is transferred from hand to hand and from mouth to mouth. This process can be a substantial source of cross infection [3]. Although some cafés offer disposable hose tips or a disposable hose (ironically called "the hygienic hose"), most narghile smokers use the regular hose but not the hygienic hose (Figure-1). This could be due to the extra cost of the hygienic hose, consequently it is only offered by the elite cafés. In addition, many people are not aware of its availability, or simply they dislike it because it does not provide a smooth smoking technique in contrast to the conventional hose, which has a relatively large diameter that demands a less suction power (Figure-1). Another disposable part that is more popular than the hygienic hose is the disposable plastic tip which is inserted in the mouth. This part called the "Mabsam" (mouth in Arabic) is usually made of plastic (Figure-1). The potential health hazards of the prolonged use of plastic "Mabsams" has not been investigated yet, and should not be overlooked, particularly that it may become heated during the smoking procedure, if the liquid in the water tanks does not adequately cool the smoke. The water chamber in narghile is capable of absorbing part of the toxic chemicals associated with tobacco burning, however, there is no guarantee that this water is frequently changed by the café staff or that the chamber is regularly cleaned. This puts in doubt the ability of this water chamber to remove away toxic materials from the smoke.

**Figure 2:**
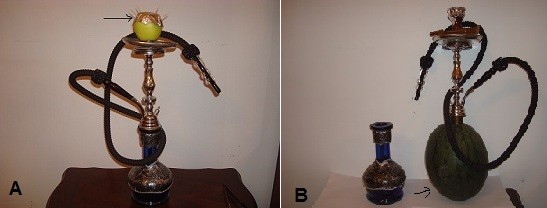
**The use of some fruits in replacement of narghile parts is preferred by many female café customers**. A: Ceramic narghile head is replaced with an apple. B: A watermelon is used to replace the water tank of the narghile.

Another debatable issue of NS is the head of the narghile which is usually made of ceramic. At cafés some ladies request that the head of the narghile to be prepared of fruits like apple for example (Figure-2, a). The head is made of half an apple by making it hollow in the middle with a hole prepared at the base to allow for smoke passage down to the water tank. Byproducts of tobacco burning into an apple with an ignited and burning charcoal at the top have not been examined yet nor have their adverse health effects been investigated.

In some of the Middle Eastern countries, the water tank is replaced with a watermelon, a melon, or even a coconut that is made hollow from the inside to accommodate the water (Figure-2, b). The effect of passing tobacco smoke onto the insides of these fruits is yet to be explored.

## Discussion

Empowerment of women is an important trend affecting most Arabic countries. Consequently, Arabic women can now enjoy a number of privileges like higher education, better career opportunities and as a result an increased spending power. Numbers of women judges, ministers, members of parliament... etc. are on the rise. Unfortunately, many customers of the elite cafés serving narghile in many Arabic cities are actually highly educated, successful women from better-off families. This image contributes to encouraging NS among younger generations particularly female adolescents.

Social pressure and antismoking campaigns are being directed against cigarette smoking and have been relatively successful in limiting cigarette smoking by females in Arabic countries. Furthermore, laws banning smoking in public premises have contributed to the reduced numbers of cigarette smokers. On the other hand, there are no laws forcing narghile café owners to check the age of their customers or to monitor the "hygiene" of their narghiles.

Family role in combating the habit of NS seems to be lacking. On the contrary, a family member like a father, a sister, a brother, or a mother could be the one introducing NS to the young adolescent female relative. The lack of awareness on narghile hazards added to the absent role model of mothers smoking narghile contributes largely to encouraging narghile use among young females.

Globalization in the era of social networking and information technology has minimized the role of the family and transformed it into a rather permissive and more lenient attitude. Young people in the Arab world are no longer compliant as they used to be, and they would like to argue on many issues including social habits like the narghile.

The World Health Organization in collaboration with the ministries of health play a major role in delivering educational programs not only to the school students but also to the parents who should play a more active role in understanding and communicating with their children.

Since peer pressure is a well-recognized aspect as far as narghile smoking is concerned, it is important to involve young generation in the fighting of the narghile epidemic.

On the other hand, governments can, and should, play an active role in regulating the work of cafés serving narghile, whereby regular periodic checks are performed on the café facilities, and when necessary, the confiscation of instruments that do not comply with medical and hygienic standards. Furthermore, public should be made aware of the availability of disposable parts of the narghile like hoses and tips to reduce the communicable diseases.

To that end, it is of prime importance that research is established to investigate whether the "hygienic hoses" and the disposable "mabsams" are as hygienic as they are thought to be

## Conclusion

In conclusion, data pertaining to the health hazards of narghile has to be distributed outside scientific journals; it is the right of the public to be aware of the risk of NS. Ministries of health in Arabic countries should play a more active role in licensing, and monitoring narghile cafés.

## Competing interests

The authors declare that they have no competing interests.

## Authors' contributions

ND and OA have contributed equally to writing this commentary. Both of them have read and approved the final manuscript.

## References

[B1] Maziak W Fouad FM Asfar T Hammal F Bachir EM Rastam S Eissenberg T Ward KD Prevalence and characteristics of narghile smoking among university students in Syria Int J Tuberc Lung Dis 2004 8 7 882 889 15260281

[B2] Azab M Khabour OF Alkaraki AK Eissenberg T Alzoubi KH Primack BA Water pipe tobacco smoking among university students in Jordan Nicotine Tob Res 2010 12 6 606 612 10.1093/ntr/ntq055 20418383 PMC2878728

[B3] Dar-Odeh NS Bakri FG Al-Omiri MK Al-Mashni HM Eimar HA Khraisat AS Abu-Hammad SM Dudeen AA Abdallah MN Alkilani SM Al-Shami L Abu-Hammad OA Narghile (water pipe) smoking among university students in Jordan: prevalence, pattern and beliefs Harm Reduct J 2010 7 10 10.1186/1477-7517-7-10 20497563 PMC2893172

[B4] Korn L Magnezi R Cigarette and nargila (water pipe) use among Israeli Arab high school students: prevalence and determinants of tobacco smoking ScientificWorldJournal 2008 8 517 525 18516473 10.1100/tsw.2008.71 PMC5848745

[B5] Labib N Radwan G Mikhail N Mohamed MK Setouhy ME Loffredo C Israel E Comparison of cigarette and water pipe smoking among female university students in Egypt Nicotine Tob Res 2007 9 5 591 596 10.1080/14622200701239696 17454715

[B6] Bahar Z Okcay H Ozbicakci S Beser A Ustun B Ozturk M The effects of Islam and traditional practices on women's health and reproduction Nurs Ethics 2005 12 6 557 570 10.1191/0969733005ne826oa 16312085

[B7] Islam SM Johnson CA Correlates of smoking behavior among Muslim Arab-American adolescents Ethn Health 2003 8 4 319 337 10.1080/13557850310001631722 14660124

[B8] Nuwayhid IA Yamout B Azar G Kambris MA Narghile (hubble-bubble) smoking, low birth weight, and other pregnancy outcomes Am J Epidemiol 1998 148 4 375 383 9717882 10.1093/oxfordjournals.aje.a009656

[B9] Soweid RA Lebanon: water pipe line to youth Tob Control 2005 14 6 363 364 16319352 PMC1748117

[B10] Tamim H Terro A Kassem H Ghazi A Khamis TA Hay MM Musharrafieh U Tobacco use by university students, Lebanon, 2001 Addiction 2003 98 7 933 939 10.1046/j.1360-0443.2003.00413.x 12814499

[B11] Chaaya M Jabbour S El-Roueiheb Z Chemaitelly H Knowledge, attitudes, and practices of argileh (water pipe or hubble-bubble) and cigarette smoking among pregnant women in Lebanon Addict Behav 2004 29 9 1821 1831 10.1016/j.addbeh.2004.04.008 15530724

[B12] Moh'd Al-Mulla A AHS Al-Lawati J Al Nasser S Ali Abdel Rahman S Almutawa A ASB Al-Bedah AM Al-Rabeah AM Ali Bahaj A El-Awa F CW JN Asma S Prevalence of tobacco use among students aged 13-15 years in Health Ministers' Council/Gulf Cooperation Council Member States, 2001-2004 J Sch Health 2008 78 6 337 343 10.1111/j.1746-1561.2008.00311.x 18489467

[B13] Al-Turki YA Smoking habits among medical students in Central Saudi Arabia Saudi Med J 2006 27 5 700 703 16680263

[B14] Mandil A BinSaeed A Ahmad S Al-Dabbagh R Alsaadi M Khan M Smoking among university students: a gender analysis J Infect Public Health 2010 3 4 179 187 10.1016/j.jiph.2010.10.003 21126722

[B15] Harrabi I Maaloul JM Gaha R Kebaili R Maziak W Ghannem H Comparison of cigarette and waterpipe smoking among pupils in the urban area of Sousse, Tunisia Tunis Med 2010 88 7 470 473 20582881

[B16] Al-Safi SA AN Albalas MA Al-Doghim I Aboul-Enein FH Does shisha smoking affect blood pressure and heart rate? J Public Health 2009 17 2 121 126 10.1007/s10389-008-0220-y

[B17] Hakim F Hellou E Goldbart A Katz R Bentur Y Bentur L The acute effects of water pipe smoking on the cardio- respiratory system Chest 2010 10.1378/chest.10-1833 21030492

[B18] Mohammad Y Kakah M Chronic respiratory effect of narguileh smoking compared with cigarette smoking in women from the East Mediterranean region Int J Chron Obstruct Pulmon Dis 2008 3 3 405 414 18990968 10.2147/copd.s1347 PMC2629988

[B19] Al-Kubati M Al-Kubati AS al'Absi M Fiser B The short-term effect of water-pipe smoking on the baroreflex control of heart rate in normotensives Auton Neurosci 2006 126-127 146 149 16716761 10.1016/j.autneu.2006.03.007

[B20] Nafae A Misra SP Dhar SN Shah SN Bronchogenic carcinoma in Kashmir Valley Indian J Chest Dis 1973 15 4 285 295 4781266

[B21] Gunaid AA Sumairi AA Shidrawi RG al-Hanaki A al-Haimi M al-Absi S al-Hureibi MA Qirbi AA al-Awlagi S el-Guneid AM Oesophageal and gastric carcinoma in the Republic of Yemen Br J Cancer 1995 71 2 409 410 10.1038/bjc.1995.83 7841062 PMC2033609

[B22] Bedwani R el-Khwsky F Renganathan E Braga C Abu Seif HH Abul Azm T Zaki A Franceschi S Boffetta P La Vecchia C Epidemiology of bladder cancer in Alexandria, Egypt: tobacco smoking Int J Cancer 1997 73 1 64 67 10.1002/(SICI)1097-0215(19970926)73:1<64::AID-IJC11>3.0.CO;2-5 9334811

[B23] Lo AC Soliman AS El-Ghawalby N Abdel-Wahab M Fathy O Khaled HM Omar S Hamilton SR Greenson JK Abbruzzese JL Lifestyle, occupational, and reproductive factors in relation to pancreatic cancer risk Pancreas 2007 35 2 120 129 10.1097/mpa.0b013e318053e7d3 17632317

[B24] Ben Saad H The narghile and its effects on health. Part II: the effects of the narghile on health] Rev Pneumol Clin 2010 66 2 132 144 10.1016/j.pneumo.2009.08.011 20413049

[B25] Dar-Odeh NS Abu-Hammad OA Narghile smoking and its adverse health consequences: a literature review Br Dent J 2009 206 11 571 573 10.1038/sj.bdj.2009.475 19521371

[B26] Urkin J Ochaion R Peleg A Hubble bubble equals trouble: the hazards of water pipe smoking ScientificWorldJournal 2006 6 1990 1997 17369998 10.1100/tsw.2006.332 PMC5917204

[B27] Maziak W Ward KD Afifi Soweid RA Eissenberg T Tobacco smoking using a waterpipe: a re-emerging strain in a global epidemic Tob Control 2004 13 4 327 333 10.1136/tc.2004.008169 15564614 PMC1747964

[B28] McDowell JD An overview of epidemiology and common risk factors for oral squamous cell carcinoma Otolaryngol Clin North Am 2006 39 2 277 294 10.1016/j.otc.2005.11.012 16580911

